# The Case for Using Evidence-Based Guidelines in Setting Hospital and Public Health Policy

**DOI:** 10.3389/fsurg.2016.00020

**Published:** 2016-03-29

**Authors:** Ross H. Francis, Jordan A. Mudery, Phi Tran, Carol Howe, Abraham Jacob

**Affiliations:** ^1^Department of Otolaryngology – Head and Neck Surgery, The University of Arizona College of Medicine, Tucson, AZ, USA; ^2^Arizona Health Sciences Library, The University of Arizona College of Medicine, Tucson, AZ, USA; ^3^Department of Otolaryngology – Head and Neck Surgery, The University of Arizona Ear Institute, The University of Arizona College of Medicine, Tucson, AZ, USA; ^4^The University of Arizona Cancer Center, The University of Arizona Bio5 Institute, Tucson, AZ, USA

**Keywords:** surgical site infections, evidence-based medicine, operating room, personal items, public health policy

## Abstract

**Objective:**

Hospital systems and regulating agencies enforce strict guidelines barring personal items from entering the operating room (OR) – touting surgical site infections (SSIs) and patient safety as the rationale. We sought to determine whether or not evidence supporting this recommendation exists by reviewing available literature.

**Background data:**

Rules and guidelines that are not evidence based may lead to increased hospital expenses and limitations on healthcare provider autonomy.

**Methods:**

PubMed, Embase, Scopus, Cochrane Library, Web of Science, and CINAHL were searched in order to find articles that correlated personal items in the OR to documented SSIs. Articles that satisfied the following criteria were included: (1) studies looking at personal items in the OR, such as handbags, purses, badges, pagers, backpacks, jewelry phones, and eyeglasses, but not just OR equipment; and (2) the primary outcome measure was infection at the surgical site.

**Results:**

Seventeen articles met inclusion criteria and were evaluated. Of the 17, the majority did not determine if personal items increased risk for SSIs. Only one article examined the correlation between a personal item near the operative site and SSI, concluding that wedding rings worn in the OR had no impact on SSIs. Most studies examined colonization rates on personal items as potential infection risk; however, no personal items were causally linked to SSI in any of these studies.

**Conclusion:**

There is no objective evidence to suggest that personal items in the OR increase risk for SSIs.

## Introduction

Evidenced-based medicine (EBM) encourages use of well-designed research to optimize decision-making. Initially applied to individuals, EBM is now increasingly utilized for healthcare policy design (evidenced-based practice policies) (1). The highest levels of medical/surgical evidence come from randomized clinical trials or meta-analyses/systematic reviews of randomized trials; case-control trials, prospective studies, and retrospective analyses provide decreasing epistemological strength in EBM. When data are not available, regulations are typically based on “expert opinion,” which the Oxford Centre for Evidence Based Medicine considers the lowest form of EBM (2). Any guideline imposed on a hospital or its staff that creates inconvenience, increases cost, impacts efficiency, decreases patient or healthcare provider autonomy, and/or potentially increases risk for patient harm (no matter how obscure the scenario) should be based on solid experimental or epidemiological data as well as the absence of equally safe and effective alternatives.

With the hope of maintaining operating room (OR) sterility, infection control regulations increasingly limit the entry of personal items into operating suites. Most personal items (i.e., handbags, purses, wallets, pens, badges, pagers, backpacks, keys, and phones) are not stored near the sterile field but are now singled out for exclusion from the OR. Yet, there are multiple unsterile pieces of equipment that enter and/or stay in the OR, which seem exempt from the same rules. For example, patients are transported to the OR on beds; patient eyeglasses or hearing aids may remain on them until anesthesia is about to be induced; multiple desktop computers and land-based phones are present in an OR suite; and health care providers wear corrective eyeglasses or loupes for magnification during surgery.

Personal items belonging to physicians and other OR staff typically allow them to be more accessible and efficient in the OR. Many surgeons keep their cell phones, pagers, and work documents in their bags, which, if nearby, can be conveniently accessed to confirm patient information from their notes or for dealing with an emergency (3, 4). Valuables are also kept in these bags so as to prevent loss or theft. Physician offices and locker rooms can be remote from the OR; therefore, back-and-forth travel to these areas between OR cases can limit productivity. By banning personal items from the OR, an inconvenience is placed on surgeons and other staff that interrupts workflow and potentially decreases physician autonomy. In this study, we sought to determine whether there is evidence supporting the recommendation that personal items be barred from the OR due to an increased risk for surgical site infections (SSIs). We also discuss the potential adverse impact of instituting hospital or public health policy that is not based in evidence.

## Methods

A review of the literature was designed and performed using methods specified in the *PRISMA statement for reporting systematic reviews and meta-analyses* (5). Both controlled vocabulary terms (e.g., MeSH) and key words were utilized to search the following databases for studies looking at the incidence or risk of infection in OR settings in association with the presence of ­personal items: PubMed/MEDLINE (1946–2015); Elsevier/Embase (1947–2015); Elsevier/Scopus (1823–2015); Wiley/Cochrane Library (1898–2015); Thomson-Reuters/Web of Science (1898–2015); and EBSCO/CINAHL (1937–2015). Literature searches were completed on February 20, 2015. The complete Pubmed/MEDLINE search strategy, analogous to the other database searches, is available in Datasheet S1 in Supplementary Material. References and citations from the articles selected from the database searches were also screened. Inclusion criteria initially were (1) Items of interest in the study were personal items (e.g., eyeglasses, cell phones, pagers, jewelry, backpacks, etc.) and not surgical equipment or surgery-related items and (2) outcome measures were to include infection at site of surgery and not generalized infections, such as pneumonia or urinary tract infections. However, because there were essentially no data about actual infection, studies looking at colonization rates of personal items were accepted as surrogate indicators of potential infection. No publication date limits were applied. Titles and abstracts of retrieved references were initially screened for relevance by two independent reviewers (Phi Tran and Ross H. Francis). In case of disagreements, a third reviewer (Jordan A. Mudery) cast the deciding vote. The full texts of the articles, thus, selected were then further analyzed to see if they met inclusion criteria. The senior author, Abraham Jacob, approved all articles selected for final analysis. Letters, case studies, review articles, conference proceedings, non-peer-reviewed articles, and clinical trials were excluded, as were articles in languages other than English.

## Results

**Figure 1:**
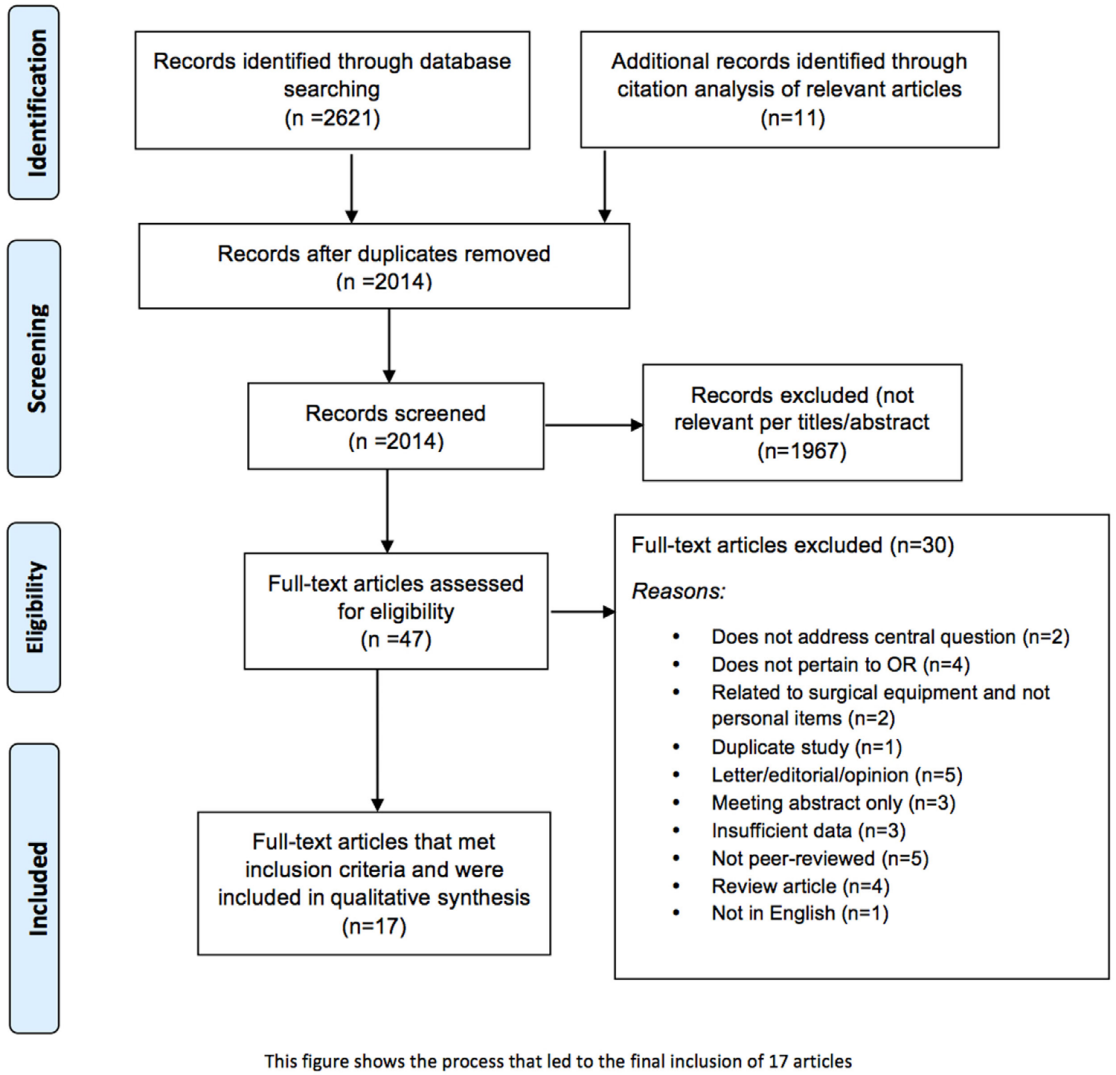
**Flowchart of the process of literature search and extraction of studies meeting the inclusion criteria**.

An initial search of selected databases revealed 2621 articles for consideration. Citation tracking of the most relevant studies revealed an additional 11 articles. Of the 2014 articles that remained after duplicates were removed, 1967 were excluded because of irrelevance to the topic (Figure 1). Strict inclusion criteria, as outlined above, were applied to the full text of 47 articles. Of these, 17 met inclusion criteria. Several of the articles reported on experiments that were performed in the OR that either involved bacterial counts and/or measured the rate of SSIs; but almost none of the articles correlated the results with SSIs. Seventeen studies, as seen in Table 1, included information on the presence of bacteria on personal items, such as cell phones, pagers, eyeglasses, and key cards, but only Stein and Pankovich-Wargula (6), in their level III retrospective cohort study, did further analysis on whether their objects of interest, wedding rings worn under surgical gloves, were actually associated with SSIs (7). A single surgeon performed a total of 2127 operations, 987 in the first 2 years without a wedding ring, and 1140 performed during subsequent years with a wedding ring worn under surgical gloves. The same scrub techniques were used in both the control (no ring) and experimental (ring) stages of the analysis, and the authors concluded that there was no relationship between wearing a plain wedding band under the surgical glove and an increased number of surgical infections (7). This was the only article found in which the authors directly investigated a potentially causal link between personal items in the OR and SSIs.

**Table 1:** **Articles that met inclusion criteria**.

Study title	Reference	Year	Study design	Sample size	Intent of study
Wedding rings are not a significant source of bacterial contamination following surgical scrubbing	Al-Allak et al. (8)	2008	Observational study	19	Do wedding rings increase infection risk?
Surveillance of bacterial colonization in operating rooms	Alexander et al. (9)	2013	Observational study	Thirty-three operating rooms, 517 samples	Determine sources of surgical infections
Theater shoes – a link in the common pathway of postoperative wound infection?	Amirfeyz et al. (10)	2007	Observational study	120	Assess bacterial contamination of OR shoes at beginning and end of day; compare results with outdoor footwear
Effect of jewelry on surface bacterial counts of operating theaters	Bartlett et al. (11)	2002	Observational study	20, with three jewelry types	Does jewelry increase bacterial count?
A socio-technical, probabilistic risk assessment model for surgical site infections in ambulatory surgery centers	Bish et al. (12)	2014	Probabilistic risk assessment model	a*	Risk assessment of SSIs in ambulatory surgery centers
Infection risk from surgeons’ eyeglasses	Butt et al. (13)	2012	Observational study	40	Check bacteria on eyeglasses
Rings and watches: should they be removed prior to operative dental procedures	Field et al. (14)	1996	Comparative study	Forty volunteers (20 dental surgeons, 20 non-clinical staff)	Compare bacteria of rings and watches from dental surgeons to non-clinical staff
Bacterial contamination of anesthetists’ hands by personal mobile phone and fixed phone use in the operating theater	Jeske et al. (15)	2007	Comparative study	40	Check contamination from cell phones vs. fixed phones on anesthetists hands after use
Should finger rings be removed prior to scrubbing for theater?	Kelsall et al. (16)	2006	Observational study	Thirty-two subjects, 18 samples from each	Does finger ring increase contamination on skin before and after scrubbing?
Mobile phones in clinical practice: reducing the risk of bacterial contamination	Mark et al. (3)	2014	Observational study	Swabbed 50 mobile phones, 150 healthcare workers	Do mobile phones harbor pathogens that could increase nosocomial infections?
Investigation of cell phones as a potential source of bacterial contamination in the operating room	Shakir et al. (4)	2015	Observational study	53	Do phones have pathogenic bacteria that could cause SSI?
Are we aware how contaminated our mobile phones with pathogenic bacteria	Singh et al. (17)	2011	Comparative study	Hundred hands and mobile phones	Do phones have pathogenic bacteria?
The dilemma of the wedding band	Stein and Pankovich-Wargula (7)	2009	Level III retrospective cohort study	2127 surgeries	Do wedding rings increase infection rates?
Security swipe cards and scanners are a potential reservoir for hospital-acquired infection	Sultan et al. (18)	2009	Observational study	Forty-five surgeons	Do cards and scanners carry pathogenic bacteria?
Are we aware how contaminated our mobile phones with nosocomial pathogens?	Ulger et al. (19)	2009	Comparative study	Two hundred healthcare workers hands and cell phones	Do cell phones have pathogenic bacteria on them?
Comparison of bacterial counts in glove juice of surgeons wearing smooth band rings vs. those without rings	Waterman et al. (6)	2006	Comparative study	20	Do rings increase infection risk?
Influence of rings on the efficacy of hand sanitization and residual bacterial contamination	Wongworawat and Jones (20)	2007	Comparative study; RCT	Sixty perioperative staff and med students	Do rings increase infection risk?
Procedure	Results	Weaknesses	Author Recommendations		
Cultured right and left hand after scrubbing in, also cultured ring	No significant difference between right and left hand. Minimal growth overall	Small sample size	Do not need to remove wedding ring for surgery		
Took samples from surfaces within operating rooms, including shoes, masks, hats	Floors, shoes, and hats had some bacterial growth. Inside of masks had the most, so mask leakage is a concern	Do these cultures directly cause surgical site infections? Why do some cause SSI and some don’t?	Wear masks and hair covers		
Randomized microbiological swab and culture	Pathogenic bacteria seen in SSIs seen in all shoe groups. Outdoor footwear most contaminated	Study does not show degree of wound contamination originating from shoes	Dedicated OR shoes and routine floor washing controls the level of shoe contamination		
Studied nose, ear, and finger rings	Finger, nose, and ear rings all increased surface bacterial count *in situ*, and especially after removal	Small sample size	Nose and ear rings should be left in but covered. Further testing recommended		
Statistical analysis	Critical components of “failure to protect patient” included several failure risk points related to skin preparation, antibiotic administration, etc.	No actual study performed, just statistical analysis	Decrease non-compliance in terms of “failure to protect patient.”		
Swabbed and incubated samples from eyeglasses	Eyeglasses are a source of SSI, all glasses grew Staph	No proof that eyeglasses are the cause of infection themselves	Disinfect eyeglasses, or wear some sort of protection over them		
Swabbed four different sites of each participant and plated for growth	Few qualitative differences were found between the microflora in the two volunteer groups	Small sample size	Bacterial flora isolated from volunteers do not commonly cause oral infections but could cause a threat to immunocompromised patients		
Had anesthetists use phones, then swabbed hands and cultured	Cell phones and fixed phones similar contamination frequency	No direct link of phone contamination to SSIs	Be careful with using cell phones in OR, as they are usually used closer to patient		
Swabbed fingers before and after scrubbing, once with ring taken off	Increased bacterial contamination beneath rings, even after scrubbing	No direct link to SSIs	Remove finger rings prior to scrubbing for surgery		
Swabbed phones, asked healthcare workers questionnaire on how much they use their phone	Cell phones safe as long as hands are clean	No real connection to SSIs	Adhere to hand cleaning procedures		
Swabbed phones with ATP bioluminescence	Cell phones has pathogenic bacteria on them, but were better once disinfected	No connection to SSIs	Routinely disinfect phones (more than once per week)		
Swabbed hands and phones	Hands and phones were contaminated	No connection to SSIs, did not test disinfecting them	Phones could be source of SSIs		
Looked at 2127 surgeries over 4 years. First two without, last two with wedding ring. Looked at how many had SSI	No correlation between wedding ring and SSI	Only one surgeon, knew he was a part of a study, lots of bias	Wedding rings are okay in the OR if scrubbed properly		
Cultured security cards and did questionnaire regarding how much surgeons used them, swabbed scanners too	Keeping card in pocket or wallet increases amount of pathogenic bacteria, scanners also had bacteria	Assumes that all surgeons bring swipe or ID badges into OR	Disinfect cards and card scanners		
Cultured healthcare workers hands and cell phones	Phones and cell phones had pathogenic bacteria on them	No analysis on how bacteria on hands and cell phones is transferred to wound	Disinfect hands and cell phones		
Cultured “juice” from gloves that were worn by vet students with and without rings, pre and post scrubbing	No significant difference between ringed and non-ringed	No assessment of glove tears from rings	Wearing rings should be fine during surgery		
Cultured “glove juice” from ringed hand and non-ringed hand	No significant difference between ringed and non-ringed	Is glove juice method appropriate for collecting bacteria?	Wearing rings should be fine during surgery		

a*: in the paper by Bish et al., no sample size is given since the paper is theoretical in nature and does not use any subjects for experimentation.

The vast majority of studies retrieved during this review examined personal items as potential sources for contamination in the OR, but did not correlate this contamination with SSIs. One of the papers (13) looked at eyeglasses, which can harbor significant quantities of skin flora, and hypothesized that contamination of the wound could occur from the presence of the eyeglasses in the OR. Potential scenarios for such contamination included: (1) eyeglasses falling from the surgeon into the wound, (2) the surgeon touching the glasses during the operation and then touching the wound, (3) saline solution droplets (during irrigation) splashing onto glasses and back into the wound, and finally (4) the eyeglasses contaminating the surgical site via airborne bacteria. The authors recommended that surgeons disinfect their eyewear or use additional personal protective equipment; however, all concerns were theoretical, and there was no research performed to show an increase in SSI from wearing eyeglasses. As far as we are aware, there are no restrictions currently in place that limit use of personal eyeglasses in the OR.

Jeske et al. (15) compared bacteria on mobile phones with those on fixed phones in the OR. The purpose was to compare the role phones might have in the spread of bacteria to the hands of physicians. Human pathogenic and non-human pathogenic bacteria were found on both mobile and fixed phones in similar quantities. The researchers concluded that there might be a potential risk of SSI from using phones in the OR; but importantly, this article did not quantify SSIs or investigate whether phone use had actually contributed to any observed infections. As a practical matter, surgeons would re-glove or re-scrub their hands following use of a phone if there was any direct contact with his/her gloves; therefore, one would not expect any increased risk of SSI.

Singh et al. (17) looked at bacteria from both the hands and mobile phones of healthcare workers inside the OR and intensive care unit. Similar types and counts of bacteria were found on hands and phones, including various bacterial pathogens, *Escherichia coli*, *Klebsiella*, *Staphylococcus*, *Streptococcus*, and *Proteus*. They concluded that both hands and mobile phones harbor bacteria and may be potential sources of nosocomial infections. They recommend that phones be disinfected regularly and often; however, these recommendations were again based on theoretical concerns, as this study did not specifically determine whether phones were causally linked to SSI.

Alexander et al. (9) evaluated potential sources of microbial contamination in the OR by randomly taking 517 samples of various surfaces in 33 ORs. They found that OR equipment that was regularly decontaminated, including anesthesia carts, operating tables, and the floors, contained enough bacteria to grow a small number of colonies. Tops of shoes and personal hats had much higher rates of bacterial contamination. No data were presented to show that OR caps or shoes increased SSI rates. Amirfeyz et al. (10) examined the difference in bacterial contamination between dedicated OR shoes vs. shoes worn from outside the OR. The authors felt that shoes worn within the OR might contribute to air contamination. While outdoor shoes were shown to have more bacterial contamination, the study failed to show an objective correlation to rates of patient infection. They concluded that current ORs that are equipped with laminar flow and high air turnover rates should reduce this risk.

Finally, Sultan et al. (18) examined whether or not security swipe cards harbored pathogenic bacteria. Security swipe cards are used frequently by hospital staff throughout the day to gain access to different working areas of the hospital, and are often worn close to various patients. The researchers swabbed cards from physicians to identify and measure bacterial load. All cards showed contamination with bacteria, and 21% of the cards were found to have pathogenic bacteria. The authors concluded that the physicians’ swipe cards are often contaminated, but did not investigate any correlation with SSIs. Interestingly, badges are typically not restricted in ORs and are often worn on the surgeon’s chest or hip – closer to the operative field than many other personal items, such as bags or purses, which are currently barred from the OR.

Ultimately, the findings of the results demonstrate only one paper that analyzed the associations between the introduction of personal items into the OR and SSIs. The other articles, while analyzing bacterial counts on various personal items, fail to address the association, if any, to SSI rates.

## Discussion

This paper aimed to determine whether there was objective, high-quality evidence linking personal items in the OR to an increased risk of SSIs. Our group initially identified over 2600 citations; however, after a two phase screening process, only 17 articles met inclusion criteria. Of these, only one [on wedding rings by Stein and Pankovich-Wargula (7)] actually evaluated SSIs as an outcome measure in their study. These authors determined that wearing a wedding band under surgical gloves during surgery did not increase risk of SSIs. Several other studies looked at bacterial contamination as a surrogate measure of infection risk (Table 1). As would be expected, bacteria were found on all items – with surgical badges standing out prominently, as did various surfaces throughout the OR. However, these bacterial contamination studies did not causally link contamination to an objectively increased risk for infection. Therefore, we conclude from our review that there is no direct evidence linking common personal items in the OR to an increased risk for surgical site infections.

The concept that personal items may serve as vectors of bacterial transmission in hospital settings is hardly new. Throughout the world, regulations have been placed on medical staff to limit their use of personal items. An anecdotal example is related by Dr. Henry Marsh, the author of *Do No Harm: Stories of Life, Death, and Brain Surgery*. In this book, he discusses the strict rules of dress imposed by the administrators of Britain’s National Health Service. Marsh speaks about being stalked by a regulating bureaucrat of the hospital who continually asked him to remove his wristwatch while visiting patients (21). This was a source of frustration for him then, and such frustrations remain today – many surgeons being cited by surveyors from regulating agencies for bringing personal items to healthcare settings, including the OR.

For a SSI to occur, bacteria must actually enter the surgical wound, and research has shown that at minimum, 10^5^ microorganisms per gram of tissue are required for a SSI to occur (22). Amirfeyz et al. suggest that a major risk factor for SSI is potentially contaminated air within the OR (10), while Altemeier et al. proposes that most SSIs come from natural flora of the patient’s skin, mucous membranes, or hollow organs (23). The major source of airborne bacteria within the OR is thought to be shed skin squames (24), not equipment. In fact, surgical site infections are directly correlated to the number of people inside the OR and their movements (25). Proper prepping, draping, and surgical technique reduce endogenous patient flora from entering surgical sites, while scrubbing of hands by the surgeon, appropriate patient draping, personal protective equipment covering surgeon skin, and technologies in the OR controlling air-flow help to prevent airborne bacteria from entering the sterile field. ORs are equipped with positive pressure ventilation systems that move air away from the sterile field. In fact, most OR ventilation systems are designed to produce a minimum of about 15 air changes of filtered air per hour, with air being introduced at the ceiling and exhausted near the floor (26). Some ORs are even equipped with laminar flow capability, which ensures that only particle-free air is brought near the sterile field.

Studies have shown that physicians who encounter quality-of-care constraints on autonomy (especially without evidence) feel discontent and stressed in their working environment (27–30). Furthermore, recent research suggests that when a physician is exposed to environmental stressors, not only does the physician suffer but also the healthcare environment and, ultimately, patient care suffer as well (31–38). So, while a single regulation such as no personal items in the OR might not seem significant, it can contribute to the accumulated stressors that affect every physician – potentially with long-term negative effects on physician morale, performance, co-workers, and the healthcare delivery system. The Association of American Medical Colleges estimates that by 2025, the United States will face a shortage of between 46,000 and 90,000 physicians (39). Therefore, it is imperative that we not exacerbate this impending healthcare crisis, as Landon et al. assert that dissatisfied doctors are two to three times more likely to exit the career of medicine than satisfied doctors (40). It is possible that if there was evidence that wristwatches could potentially endanger a patient, Dr. Marsh would have been much more enthusiastic about following his administrator’s guidelines. In fact, physicians as a whole are more likely to be compliant with recommendations when they were based on high-quality evidence (41–44).

## Conclusion

As expected for any object exposed to air, personal items carried into the healthcare environment are contaminated with environmental bacteria. Bacterial contamination of stationary items in ORs, patient rooms, etc. is also a reality. In an era of expanding, often onerous regulations, we sought to determine whether personal items (i.e., handbags, purses, wallets, pens, badges, pagers, backpacks, keys, earrings, necklaces, phones, and eyeglasses) brought into the OR increase risk for surgical site infection. This analysis is time appropriate as such personal items have come under scrutiny by regulators. Our systematic review found no literature supporting a causal link – suggesting that current regulations barring personal items from the OR are based on theoretical concerns or “expert opinion,” rather than objective evidence. Doctors comply with recommendations based in data; therefore, regulating agencies should support well-done, prospective clinical studies to better inform the application of new policies.

## Author Contributions

RF and JM had full access to all of the data in the study and take responsibility for the integrity of the data and the accuracy of the data analysis. Study concept and design: Drs. AJ and CH. Acquisition, analysis, or interpretation of data: all authors. Drafting of the manuscript: all authors. Critical revision of the manuscript for important intellectual content: all authors, we would also like to acknowledge Dr. Bruce Mannes M.D., Dr. David Ellsworth DPM, and Renee Cercone for reviewing our manuscript. Administrative, technical, or material support: all authors. Study supervision: AJ and CH. Conflict of Interest and Financial Disclosures: none reported.

## Conflict of Interest Statement

The authors declare that the research was conducted in the absence of any commercial or financial relationships that could be construed as a potential conflict of interest.
