# Supplementary material for: The Case for Using Evidence-Based Guidelines in Setting Hospital and Public Health Policy
Source: Front Surg. 2016 Mar 29;3:20. doi: 10.3389/fsurg.2016.00020 (PMC4810072; doi:10.3389/fsurg.2016.00020)
Supplement: Supplementary file 1 [file Data_Sheet_1.PDF]

Appendix A

PubMed Search Strategy

(((((((((handbag OR handbags OR "hand bag" OR "handbags" OR purse OR purses OR wallet OR wallets OR pen OR pens))) OR (badge OR badges) OR (pager OR pagers) OR ((("backpacks") OR "backpack"))) OR keys) OR ((("personal items") OR "personal item"))) OR (((jewelry[MeSH Terms]) OR jewelry) OR (((earrings) OR necklace OR necklaces)))) OR (("Cell Phones"[Mesh]) OR phone OR phones))) OR ("eyeglasses"[MeSH Terms] OR "eyeglasses"[All Fields] OR "spectacles"[All Fields])

AND

(((((("infection"[MeSH Terms] OR infect*[tiab])) OR contaminat*[tiab]) OR ((("Surgical Wound Infection"[Mesh]) OR "Infection Control"[Mesh]) OR "Cross Infection"[Mesh])))

AND

((((("Operating Rooms"[Mesh]) OR "Surgical Procedures, Operative"[Mesh])) OR (((operating) OR operative) OR operation)) OR ((surgical procedure) OR surgery)))
